# Glycan analysis of colorectal cancer samples reveals stage-dependent changes in CEA glycosylation patterns

**DOI:** 10.1186/s12014-018-9182-4

**Published:** 2018-03-02

**Authors:** Qianqian Zhao, Tiancheng Zhan, Zaian Deng, Qianqian Li, Yaming Liu, Shaojie Yang, Dengbo Ji, Yan Li

**Affiliations:** 1 Laboratory of Interdisciplinary Research, Institute of Biophysics, Chinese Academy of Sciences, 15 Datun Road, Chaoyang District, Beijing, 100101 China; 2 University of Chinese Academy of Sciences, Beijing, 100049 China; 3 Key Laboratory of Carcinogenesis and Translational Research (Ministry of Education), Department of Colorectal Surgery, Peking University Cancer Hospital and Institute, Beijing, 100142 China; 4 GuangDong Bio-healtech Advanced Co., Ltd., Foshan, 528000 China

**Keywords:** Colorectal cancer, Carcinoembryonic antigen, Glycosylation, Lectin microarray

## Abstract

**Background:**

Carcinoembryonic antigen (CEA) is a glycoprotein associated with colorectal cancer (CRC). While the functions of its gene and protein have been fully characterized, its post-translational modifications in the context of CRC development remain undefined.

**Methods:**

To show the correlation between the different stages of CRC development and changes in the glycosylation patterns of CEA, we analyzed CEA in tumor tissues (CEA-T) and paired tumor-adjacent normal tissues (CEA-A) from 53 colorectal cancer patients using a high-density lectin microarray containing 56 plant lectins.

**Results:**

We detected higher expression levels of fucose, mannose and Thomsen–Friedenreich antigen, and lower expression levels of *N*-acetylgalactosamine, *N*-acetylglucosamine, galactose, branched and bisecting *N*-glycans on CEA in the tumor tissues relative to the tumor-adjacent normal tissues. Furthermore, a combinatorial assessment of 9 lectins is sufficient to distinguish CRC tumor tissues from tumor-adjacent normal tissues with 83% sensitivity and ~ 90% specificity. Moreover, the levels of *N*-acetylgalactosamine, mannose, galactose, *N*-acetylglucosamine on CEA showed a downward trend after first experiencing an increase at Stage II with the stages of CRC.

**Conclusions:**

Our insights into the changing CEA glycosylation patterns and their role in the development of CRC highlight the importance of glycan variants on CEA for early clinical detection and staging of CRC.

**Electronic supplementary material:**

The online version of this article (10.1186/s12014-018-9182-4) contains supplementary material, which is available to authorized users.

## Background

Colorectal cancer (CRC) is the third most common diagnosed cancer globally [1]. CRC shows little symptoms in its early stage, resulting in regional or distant metastasis in most patients at the time of diagnosis, rendering treatment difficult [2]. Development of CRC occurs progressively, usually spanning 5–10 years. This extended timeframe provides ample opportunities for treatment, especially during the early stage (including the high-risk stage II) [3–5]. However, current screening methods are of low sensitivity and specificity [6]. Recently, genomic and proteomic studies found new candidate biomarkers for detecting the early stage of CRC, however, none has so far been tested in clinical trials [7, 8]. Therefore, a better understanding of the biology of CRC is paramount to more reliably predict, diagnose and monitor the disease, and to ultimately find efficient drug targets.

Glycosylation is one of the major post-translational modifications found in proteins. It alters protein function and plays an important role in many different biological processes, including protein–protein interactions, cell–cell recognition, adhesion and migration [9–11]. Aberrant glycosylation is associated with the occurrence and progression of various tumors [12]; it may be a result of initial oncogenic transformation, as well as a key event in induction of invasion and metastasis [13]. Changes in glycosylation patterns correlate well with the progression of colorectal cancer through its different stages, and have been found for *O*-glycans, *N*-glycans, globo-type glycosphingolipid (GLS)-glycans, sialylation, (Sialyl) Lexis antigens [14]. Importantly, changes in glycan modifications appear to occur more frequently than changes in the actual protein concentration [15, 16]. Thus, analysis of changes in glycosylation patterns associated with a particular protein should yield biomarkers relevant to effective cancer diagnosis. For instance, the core fucosylation of α-fetoprotein has recently been approved as a biomarker for the early detection of hepatocellular carcinoma (HCC), distinguishing it from chronic hepatitis and liver cirrhosis [17].

Human carcinoembryonic antigen (CEA) is the most frequently used marker for colorectal cancer screening, diagnosis and monitoring. Due to a lack of sensitivity and specificity, however, its clinical application has remained limited. CEA is a stable glycoprotein consisting of ~ 60% carbohydrate and a molecular mass of ~ 180–200 kDa. The carbohydrate side chains of CEA are highly variable, most of which being composed of mannose, galactose, *N*-acetylglucosamine, fucose and sialic acid [18]. Recently, we examined glycans in colorectal carcinoma tissue samples, and identified 61 *N*-glycoforms present on the surface of CEA. In one of our recent studies, we showed that the composition of the glycans associated with CEA displays a considerable heterogeneity [19]. Saeland et al. [20] compared CEA glycosylation patterns of normal and colorectal cancer tissues and found that Lewis X, Lewis Y, mannose and branched *N*-glycans are increased in tumor-associated CEA. However, a more detailed understanding of the changes in CEA protein surface glycans, especially with the development of CRC is still lacking. Importantly, analysis of carbohydrate expression profiles of CEA with the progression of CRC is crucial for the understanding of the biology of tumor growth, proliferation, and metastasis, and should aid the development of novel cancer biomarkers for early diagnosis of CRC.

Lectin is a protein found in both plants and animals that specifically binds glycan; because of this specific binding property, it has been used for glycan detection as a part of various techniques including affinity chromatography and lectin blots [21]. Lectin microarray technology is a rapid and high-throughput platform for analyzing glycosylation patterns of specific glycoproteins in clinical samples [22]. It allows simultaneous profiling of hundreds of lectins in a single screening of multiple biological samples [23]. Thus, lectin-based glycan detection methods provide a broad picture of the glycan structures present on proteins, and have been used to study changes of glycans in various diseases [24].

Here we set out to perform glycosylation profiling for CEA between tumor tissues (CEA-T) and tumor-adjacent normal tissues (CEA-A) using a high-density lectin microarray. We found that changes in the glycosylation patterns of CEA correlated well with CRC tumorigenesis and progression, with specific glycans being differently expressed on CEA in a stage-dependent manner.

## Methods

### Materials

Lectin microarrays were purchased from BCBIO (Guangzhou, China). Commercial standard Carcinoembryonic Antigen (CEA) was purchased from LEE BioSolutions, Inc (St. Louis, MO). Human Carcinoembryonic Antigen (CEA) ELISA Kit was purchased from Yu Ping biotechnology (Shanghai, China). Anti-Human CEA antibody was purchased from eBioscience Inc (San Diego, CA). Rabbit anti-mouse IgG-Alexa Fluor 647 conjugate was purchased form Invitrogen (Eugene, OR). The incubation chamber and holder for the lectin microarray were purchased from Whatman Schleicher and Schuell (Keene, NH). Sodium periodate was purchased from Bio-Rad Laboratories (Hercules, CA). 4-(4-*N*-maleimidophenyl) butyric acid hydrazide hydrochloride (MPBH) was from Thermo Fisher Scientific, Inc. (Rockford, IL). All other chemicals and reagents were purchased from Sigma-Aldrich (St.Louis, MO).

### Specimen

Samples and clinical information were reviewed and approved by the Institutional Review Board of Institute of Biophysics, Chinese Academy of Sciences. After obtaining signed informed consent, tumor tissues and paired tumor-adjacent normal tissues from 53 patients with colorectal carcinoma (stage I–IV) were collected from Beijing Cancer Hospital (patient information as listed in Table 1). All patients enrolled in the study had operative treatment of colorectal carcinoma and the surgical pathology report was used to confirm the diagnosis of UICC/AJCC′ stage of colorectal carcinoma. Each sample was immediately placed on the ice after procurement and stored at − 80 °C. All tissue samples were thawed less than three times prior to extraction in order to minimize variability introduced by that process [22].

**Table 1:** Detailed information of participating colorectal cancer patients

Stage	Gender M/F	Age (mean ± SD)	Other diseases (yes/no)	Adjacent tissue C_(CEA)_ = 0.25 μg/ml	Tumor tissue C_(CEA)_ = 0.25 μg/ml
I (17)	6/11	58 ± 12	10/7	17	17
II (14)	7/7	63 ± 12	6/8	14	14
III (9)	5/4	63 ± 10	5/4	9	9
IV (13)	9/4	65 ± 10	11/2	13	13

M, male; F, female; C_(CEA)_, the concentration of CEA; SD, standard deviation

### Protein extraction and determination of CEA concentration

Colorectal carcinoma tissues were quickly removed from the cryovial and washed using PBS buffers. Tissues were cut into pieces and weighed. 1 ml RIPA lysis buffer (0.15 g Tris, 0.438 g NaCl, 0.05 g NaOH, 0.5 g Sodiumdeoxycholate and 0.05 g SDS) was added to the 100 mg tissue pieces. The mixture was grinded into homogenate in a tissue grinder. All tissue homogenates were incubated and then centrifuged at 15,000*g* for 15 min, the supernatant was kept at – 80 °C. Subsequently, the concentration of CEA was determined using Human Carcinoembryonic Antigen (CEA) ELISA Kit [19].

### Lectin microarray

The lectin microarray was first blocked in 50 mM ethanolamine in borate buffer (pH 8.0) for 1 h at room temperature. The slide was then washed once in TBS with 0.1% Tween20 (TBST 0.1), followed by two washes in TBS and dried by spinning at 500 g for 5 min. Standard CEA was diluted into 100 μl using TBS buffer (for concentrations of 0, 0.1, 0.5, 1, 5, 10 μg/ml respectively). The samples were allowed to bind on lectin microarray and incubated at room temperature for 6 h. The primary antibody (mouse anti-human CEA antibody) and the secondary antibody (rabbit anti-mouse IgG-Alexa Fluor 647 conjugate) were mixed with 20 mM sodium periodate at 4 °C for 1 h in the dark to oxidize sugar groups. The oxidized glycans of antibodies were then blocked with 1 mM 4-(4-*N*-maleimidophenyl) butyric acid hydrazide hydrochloride (MPBH) for 2 h followed by 1 mM Cys-Gly dipeptide in 4 °C overnight [24]. The microarray was removed from the incubation chamber, and then 2 μg/mL oxidized mouse anti-human CEA antibody was sequentially hybridized with the microarray at 4 °C overnight. After washing, 2 μg/ml oxidized rabbit anti-mouse IgG-Alexa Fluor 647 conjugate was hybridized for 1 h with gentle shaking. After washing with TBST buffer, the microarray washed twice with water. The array was dried by spinning at 500 g for 5 min, and scanned using a LuxScan™ 10K-A scanner at 10 μm resolution. The scanning condition was set to 85 power and 850 PMT value for Cy5 channel. The scanned images were analyzed using LuxScan 3.0 software to convert to numerical format (GPR) using a homemade “GAL” files [25].

For clinical samples testing, CEA in all samples were diluted with TBS buffer into the same concentration. The same amount of CEA protein of each sample was applied to lectin microarray using the protocol described above. TBS buffer without protein was used as negative control.

### Data analysis

The mean of the foreground spot intensity and mean of the background spot intensity were used in this analysis. The signal-to-noise ratio (the mean of spot foreground intensity relative to the mean of spot background intensity) of each lectin spot was used to calculate each lectin [25]. Because each lectin was present in triplicate, the signal intensities from replicate lectin measurements within the same array were averaged (CV ≤ 30%). Each lectin microarray contains a negative control sample, the 95% confidence interval of the signal-to-noise of all lectins is (0.8, 1.2). The signal-to-noise ratio of greater or equal to 1.2 was defined as a positive signal. Any undetected signal was set to 1. All positive and negative signals of tissue samples were used in all subsequent data analysis.

Significant differences between CEA-A and CEA-T of colorectal carcinoma patients were tested using a two-tailed paired *t* test. The bars represent the mean values with standard error of mean (SEM). One-way analysis of variance (ANOVA) test was used to the differences between four stages. *P* values lower than 0.05 were considered as statistically significant.

## Results

### Sensitivity of lectin microarray

**Fig. 1:**
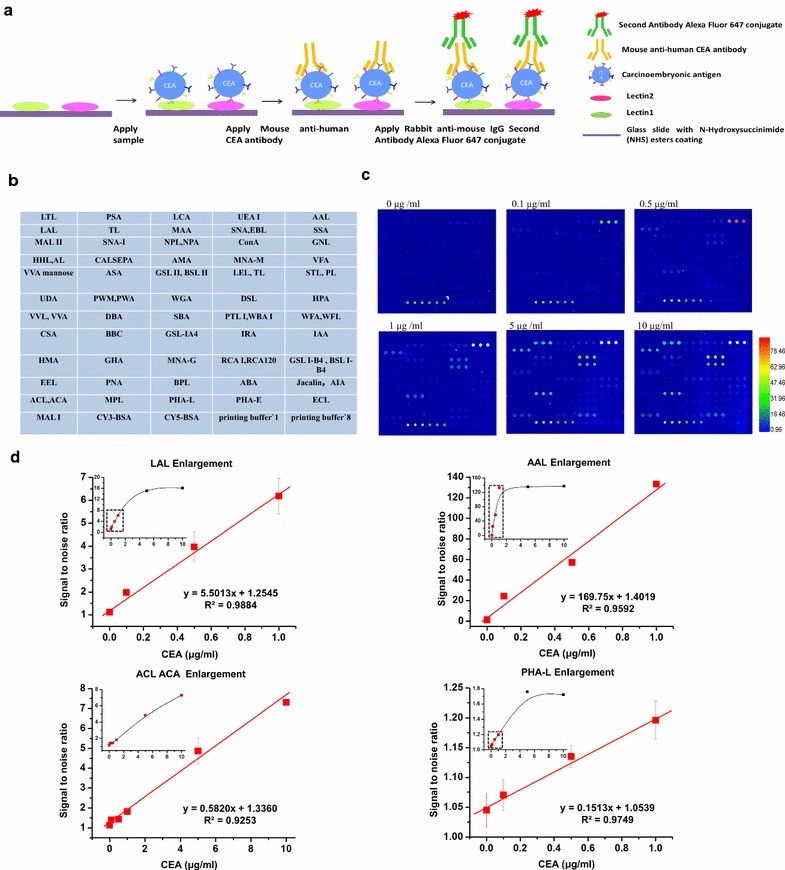
Identification of lectins interacting with purified CEA. **a** Schematic presentation of lectin microarray for CEA glycosylation analysis. Lectin 1 and 2 bind two different glycosylation patterns of CEA. **b** Design of the lectin microarray containing 56 lectins. **c** Representative lectin microarray binding patterns of six different CEA concentrations. **d** Four representative lectins bind different concentrations of CEA. The smaller diagrams show the overall changes with the increase of CEA concentration. The red points in the dashed box show expanded portions of the small diagrams. Bars represent the mean values with standard deviation (SD). CY3-BSA and CY5-BSA are positive controls. Printing buffer 1 and 8 serve as negative controls. The color bar represents corresponding signal-to-noise value

First, we established a procedure to determine CEA glycosylation patterns using a lectin microarray (Fig. 1a). As shown in Fig. 1b, the microarray contains 56 lectins, with each lectin present in triplicate. All lectins used possess diverse glycan specificities as defined in previous studies (see Additional file 1: Table S1).

Next, in order to optimize conditions for our lectin microarray procedure, we evaluated its sensitivity using commercial standard CEA purified from human liver metastases. We incubated the microarray with a series of CEA concentrations (keeping the volume of each sample at 100 μl). As shown in Additional file 2, we showed that at the highest concentration of CEA (10 μg/ml), 31 lectins specifically interacted with CEA. As shown in Fig. 1c, the signal of each detectable lectin spot increased with the concentration of CEA. In order to determine the optimal amount of clinical sample loaded, we analyzed fluorescence signal intensity for the 31 lectins as a function of CEA concentration. Four calibration curves of representative lectins are shown in Fig. 1d. Together, these results indicate that below a CEA concentration of 0.5 μg/ml, the signal-to-noise ratio of the assay is positively correlated with the concentration of CEA.

### Glycosylation pattern analysis of CEA-A and CEA-T using lectin microarray

To compare the glycosylation patterns of CEA-A and CEA-T, we collected CRC tumor and paired tumor-adjacent normal tissues from 53 patients at different stages (n = 17, stage I; n = 14 stage II; n = 9 stage III; n = 13 stage IV). The detail information of these patients was shown in Table 1. We excluded the possibility that other diseases of patients skewed experimental results (see Additional file 3). The concentrations of CEA in tissue lysates were determined using an ELISA assay; the data was shown in Additional file 4: Table S2. In order to make sure the amount of CEA in each clinical sample was identical, we adjusted CEA concentrations to 0.25 μg/ml, and maintained sample volumes at 200 μl.

**Fig. 2:**
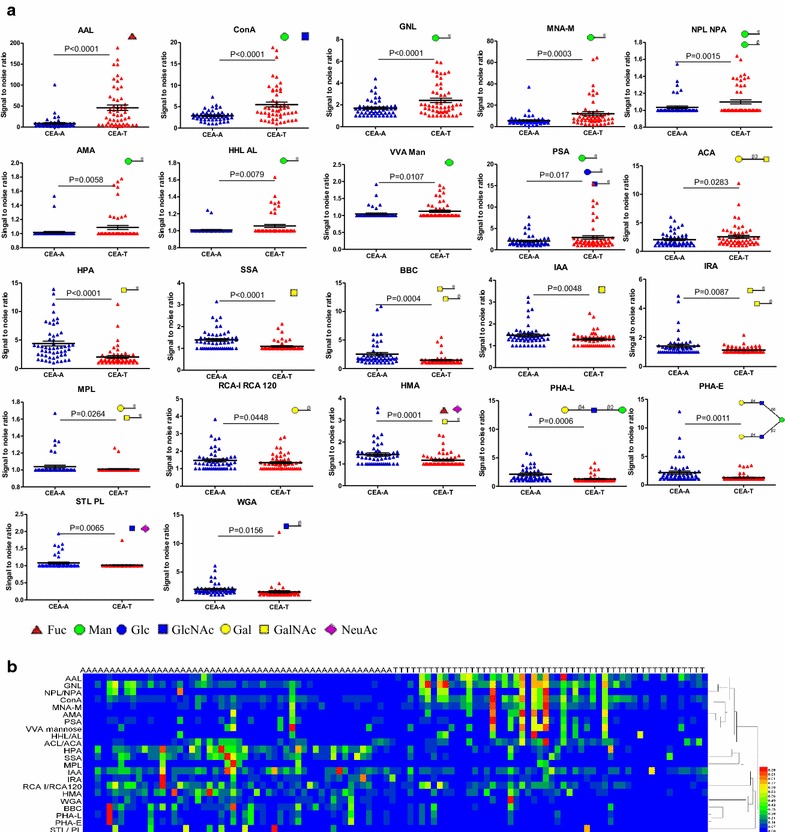
Comparison of CEA glycosylation patterns in tumor tissues and tumor-adjacent normal tissues. **a** Distribution of lectins exhibiting significant changes in binding between CEA-A and CEA-T samples. Bars represent the mean values with standard error of mean (SEM). **b** Clustered heat map of the lectin CEA-binding profiles. Lectins are indicated on the vertical axis, and samples are indicated along the horizontal axis, with A and T indicating the tumor-adjacent normal sample and tumor tissue sample, respectively. The lectin rows were grouped according to lectin-binding patterns. Each square represents the intensity of a lectin binding glycosylation pattern on CEA within a sample. To clearly show the variation between the samples, the values of signal-to-noise ratios were transformed by Min–Max normalization. [yi = xi − min (xj)/max (xj) − min (xj), (1 ≤ i ≤ n, 1 ≤ j ≤ n); Max (xj) is the maximum value of the samples, min (xj) is the minimum value]. The color bar represents the scale. CEA-A, CEA in tumor-adjacent normal tissue; CEA-T, CEA in tumor tissue; A, tumor-adjacent normal tissue sample; T, tumor tissue sample

As shown in Fig. 2a, we observed 22 lectins binding CEA had significant difference between CEA-T and CEA-A. Lectins AAL, MNA-M, Con A, GNL, AMA, HHL (AL), VVA Man, NPL (NPA), PSA and ACA binding to CEA were higher in CRC tumor tissues relative to tumor-adjacent normal tissues. The results suggest that fucose, mannose and the Thomsen–Friedenreich antigen (TF-antigen) (Core1, Galβ1-3 GalNAc-Ser/Thr) are higher expressed on CEA-T than CEA-A. Moreover, lectins HPA, SSA, BBC, IRA, IAA, MPL, RCA-C (RCA 120), HMA, PHA-L, PHA-E, STL (PL) and WGA binding to CEA were lower in CRC tumor tissues relative to tumor-adjacent normal tissues. The results suggest that *N*-acetylgalactosamine, galactose, *N*-acetylglucosamine, branched *N*-glycans and bisecting *N*-glycans are lower expressed on CEA-T than CEA-A.

To directly compare the binding of 22 lectins (see Fig. 2a for all lectins) to CEA, we generated and clustered a heat map according to lectin-binding pattern and intensity. As shown in Fig. 2b, lectin patterns showed considerable differences between CRC tissues and tumor-adjacent normal tissues. This suggests that the changes of CEA surface glycans correlated well with the presence of CRC. Different lectins with the same glycan binding specificity were clustered into one group. For example, lectins binding to mannose, such as GNL, MNA-M, NPL (NPA), AMA, VVA Man and HHL (AL), were grouped into one cluster.

**Fig. 3:**
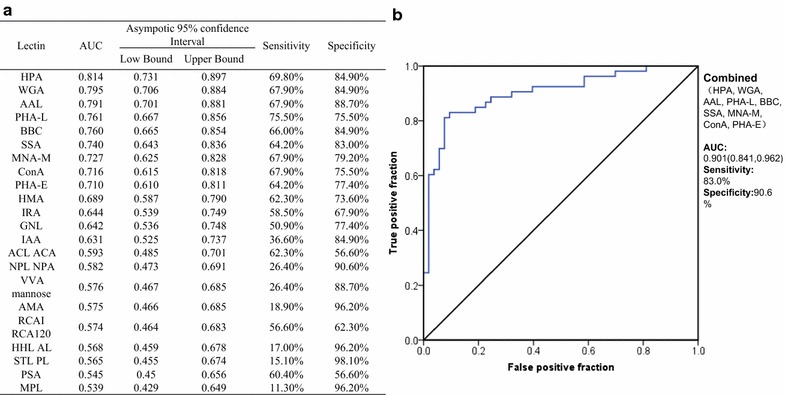
Receiver operating characteristic (ROC) curves for the discriminating tumor tissue samples from tumor-adjacent normal tissue samples using individual and combined lectins. **a** The area-under-the-curve (AUC), 95% confidence interval, sensitivity and specificity of 22 lectins alone. **b** The ROC is indicated for combination of 9 lectins

In order to evaluate the discriminative power of lectin binding to CEA-associated glycosides for the purpose of distinguishing CRC tumor tissues from tumor-adjacent normal tissues, we performed ROC analysis (Fig. 3a). Our analysis showed that the values of area-under-the-curve (AUC) of 9 lectins (HPA, WGA, AAL, PHA-L, BBC, SSA, MNA-M, Con A, PHA-E) binding to CEA were greater or equal to 0.7, which suggested that these lectins have moderate accuracy for detecting CRC tumor tissues from tumor-adjacent normal tissues. Next, we analyzed the combined panel of these 9 lectins using binary logistic regression rule. The AUC of combined lectins was 0.901 with 83% sensitivity and ~ 90% specificity (Fig. 3b). Together, these results indicate that CEA-associated glycans represent a powerful tool to distinguish CRC tumor tissues from tumor-adjacent normal tissues with sufficient sensitivity as well as specificity.

### Analysis of changes in glycosylation of CEA in colorectal carcinoma with four stages

In order to investigate the relationship between CEA concentration and CRC stages, we analyzed CEA concentration in serum and tissues with four stages of CRC using a One-way ANOVA test. As shown in Additional file 3, no significant differences in CEA concentration were observed between our biological samples, either serum, CRC tumor tissues or CRC tumor-adjacent normal tissues. Importantly, CEA concentration between samples from different CRC stages showed negligible variation.

**Fig. 4:**
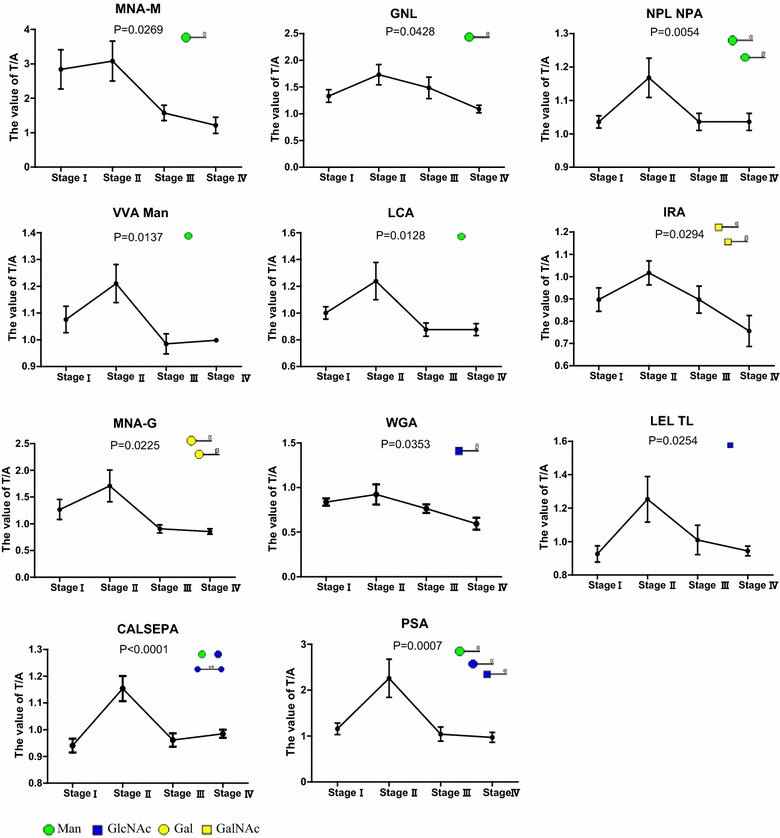
Correlations between lectins binding CEA and the stage of colorectal carcinoma. The bars represent the mean values with standard error of mean (SEM). A, tumor-adjacent normal tissue samples; T, tumor tissue samples

To evaluate whether changes in glycan patterns allow for unambiguous identification of the four stages of CRC, we performed statistical analysis of the fold changes of lectins binding to CEA between CRC tumor tissues and paired tumor-adjacent normal tissues using One-way ANOVA test. The fold changes were calculated using the signal-to-noise ratios of tumor tissue samples divided by the signal-to-noise ratios of paired tumor-adjacent normal tissue samples, indicated as the value of T/A. As shown in Fig. 4, 11 lectins bound to CEA with significant differences, indicating significant changes in CEA glycan levels of *N*-acetylgalactosamine, mannose, galactose, *N*-acetylglucosamine at different CRC stages. Interestingly, the levels of these glycosylation modifications on CEA showed a downward trend following an increase at Stage II. Together, these results indicate that CEA-associated *N*-acetylgalactosamine, mannose, galactose, *N*-acetylglucosamine increase at stage II, before falling to the levels observed at the advanced stage of CRC.

## Discussion

CEA is one of the most widely used protein biomarkers for CRC; however, its clinical use is limited due to its low sensitivity and specificity [4]. Recent studies suggested that the detection of glycan variants on a particular protein should yield more effective biomarkers than the measurement of protein concentration [24]. Our findings presented here reveal that glycosylation patterns on CEA differ significantly between CRC tumor tissues and paired tumor-adjacent normal tissues. In addition, we found that the changes of glycosylation levels on CEA correlate with the different stages of CRC.

Here, we used CRC tissues to investigate the changes of glycans on CEA rather than blood and stool samples. Although blood and stool are available and non-invasive to collect, blood is a heterogeneous mixture of proteins derived from different tissues and proteins from stool samples are degraded by the gut micro-biota [8]. Moreover, CEA is commonly detected in a number of tumors of epithelial origin such as lung adenocarcinoma except colorectal carcinoma, in some benign diseases and even in normal adult tissues [26–28], which renders identification of the source of CEA in blood and stool samples difficult. Therefore, a pivotal element of our investigation is the fact that we used tissue samples from CRC patients, ensuring that CEA protein originates in the CRC tumor itself and the detected glycosylations on CEA represent exactly their distribution in CRC patients.

Due to the large quantity of purified glycoprotein required and limited sample throughput, other traditional methods like liquid chromatography (LC), capillary electrophoresis (CE) and MALDI-TOF–MS^n^ cannot be applied to the analysis of glycan variants on CEA from individual CRC tissues [29]. Therefore, we employed lectin microarray technology to analyze the patterns of CEA glycosylation. To obtain a detailed glycan map of CEA, we applied CRC tissues lysates containing 0.5 μg CEA protein to the microarray. Apart from the small amounts of sample material required, lectin microarray technology possesses the additional advantage of global screening to identify lectins interacting glycoprotein and determining the glycosylation changes with high reproducibility and high sample throughput.

In the present study, we found that fucose (Fuc1-2,3,4) is increased in tumor-associated CEA. Fucosylated glycans can be generally divided into terminal fucosylation (giving rise to specific blood group antigens such as SLe^a^ and SLe^x^) and core fucosylation(creating a non-extendable modification) [30]. The core fucosylation (CF) of *N*-glycoproteins plays important roles in regulating protein functions during biological development [31]. Fucosylation levels increase significantly in colon cancer for *N*-glycans, *O*-glycans and globo-type glycosphingolipid (GSL) glycans [32]. In agreement with these results, we also observed that fucose(Fuc1-2,3,4) is expressed at higher levels in tumor-ssociated CEA. These elevated levels of fucosylation may be caused by upregulated fucosyltransferase VI, which was reported as a major enzyme modulating the SLe^x^ biosynthesis in colorectal cancer [33, 34].

Our results show that mannose (Man) levels are elevated in tumor-associated CEA. Previous studies showed that the cryptic and high-mannose *N*-glycans emerge in CRC tumor tissues, especially in cell lines [35, 36]. However, while the function of altered mannose in cancer progression remains unclear, it has been suggested earlier that the increase of high-mannose *N*-glycans in cancer might be the result of precursor accumulation, due to incomplete maturation during *N*-glycan biosynthesis [14, 37].

Furthermore, the Thomsen–Friedenreich antigen (TF-antigen) (Core1, Galβ1-3 GalNAc-Ser/Thr) as also increased in tumor-associated CEA. TF-antigen has been reported to be associated with metastasis [38]. Saeland et al. [20] found TF-antigen increased on MUC1 from CRC tumor tissue. Recent studies shows that Galectin-3 secreted by tumor cells binds TF-antigen on MUC1 [39, 40]. Due to the interactions between Galectin-3 and TF-antigen, clustered MUC1 on the cell surface exposes adhesion molecules, such as E-Cadherin, avoiding initiation of anoikis (suspension-induced cell death) [39]. It has been shown previously that these exposured adhesion molecules may induce interactions with endothelial cells and promote metastasis [40].

We found that the levels of branched *N*-glycans, bisecting *N*-glycans and overall *N*-acetylglucosamine (GlcNAc) are decreased in tumor-associated CEA. Enhanced *β*6GlcNAc side chain branching of N-linked structure (caused by enhanced activity of GnT-V) and counteracting *β*4GlcNAc (bisecting GlcNAc) (synthesized by GnT-V) are the most widely occurring glycosylation changes inducing malignancy [41]. GnT-V promotes metastasis, whereas GnT-III shows the opposite function [13]. Saeland et al. [20] found that branched *N*-glycans were prominently present, however, a change in bisecting *N*-glycans were not observed on tumor-associated CEA in colorectal cancer tissues. Our results are in contrast with previous studies, which may be caused by sources of material at different disease stage [14]. Therefore, further experiments are required to validate the role of branched *N*-glycans and bisecting *N*-glycans in CRC.

In addition, our analysis showed that overall *N*-acetylgalactosamin (GalNAc) was increased in tumor-associated CEA. GalNAc-type *O*-glycans are found in most transmembrane and secreted glycoproteins. The disaccharide Thomsen–Friedenreich antigen (T antigen, also known as core 1) and the mono-saccharide GalNAc (also known as Tn) and their sialylated forms (ST and STn (Neu5Acα2-6GalNAcα-O-R), respectively) result from the incomplete synthesis of *O*-glycans. Aberrant glycosylation also occurs in glycoproteins that display abnormal expression of shortened or truncated glycans during malignancy [12].

We also found that galactose (Gal) levels are lower in tumor-associated CEA. Galactosylation is involved in the regulation of immune response by modifying immunoglobulin G (IgG) properties [42]. Low levels of galactose on IgG are associated with a higher proinflammatory activity. The presence of IgG lacking galactose in early synovitis is of prognostic value for the future development of erosive rheumatoid arthritis (RA) [43]. Ruhaak et al. [44] found that galactosylation levels are reduced in the tissue samples of lung adenocarcinoma patients. Down-regulated galactose on CEA may be correlated with CRC immune response.

Importantly, our ROC analysis indicates that *N*-acetylgalactosamin, *N*-acetylglucosamine, fucose, mannose, branched *N*-glycans and bisecting *N*-glycans represent those CEA-associated glycans with the most significant changes. Thus, our analysis provides strong evidence that for clinical purposes, a combination of lectins recognizing these glycans can greatly improve the power of discrimination between CRC tumor tissue from healthy adjacent tissue, making these glycans ideal panel biomarkers for CRC diagnosis.

In our study, we observed that the levels of *N*-acetylgalactosamine, mannose, galactose, *N*-acetylglucosamine on CEA first increased at Stage II, before falling to their original values or below. This indicates that CEA glycans change dynamically with CRC development, raising the possibility that these altered glycans play transient roles in the progression of tumor. Previous studies proposed that changes in glycan patterns represent a hallmark of cancer progression [14, 45]. However, the precise molecular mechanisms for inducing such changes in CEA glycosylation levels throughout the different stages of CRC remain unclear. A number of reports found that cancer-associated changes in glycan patterns are a result of incomplete synthesis and neo-synthesis processes. More specifically, earlier studies found that incomplete synthesis occurs more often in the early stages of cancer, whereas neo-synthesis is more commonly observed in the advanced stages of cancer [12, 46]. Based on these findings, we hypothesize that levels of immature *N*-glycan (high-mannose type) and truncated *O*-glycan (*N*-acetylgalactosamine) are likely to increase in the early stages of CRC, and then decrease in the later stages of tumor development. This provides an explanation for the changes of the levels of glycosylations on CEA along with the stages of CRC. The differential expression levels of glycotransferase during cancer progression may be another reason for the changes of the levels of glycosylations on CEA with the stages of CRC. Munkley et al. [47, 48] reported that the expression of ST6GalNAc1 (the sialyltransferaseα-GalNAc α-2,6-sialyltransferase, an enzyme that catalyses the transfer of a sialic acid molecule in an α-2-6 linkage onto the Tn antigen (resulting in GalNAc1-O-serine/threonine)) was increased in primary prostate tumours and decreased in metastatic tissue relative to non-malignant prostate tissue. Further studies will be required to better understand the glycotransferase levels relevant to CEA glycan patterns.

## Conclusions

Our findings provide evidence that analysis of glycan patterns present a reliable and powerful tool for the diagnosis and staging of CRC; in addition, combinatorial analysis of specific glycan profiles possibly allows for identification of specific cancer stages. Together, temporal changes in glycan expression on marker proteins such as CEA should allow for the early detection of colorectal carcinoma, as well as lead to a better understanding of the role of CEA in the pathogenesis and progression of colorectal carcinoma.

## Additional files

**Additional file 1: Table S1.** Lectin used in this study.

**Additional file 2.** 31 lectins bind different concentrations of CEA.

**Additional file 3.** The correlation between other diseases and glycans on CEA.

**Additional file 4: Table S2.** The concentration of CEA of CRC patients; **Figure S1.** The relationship between CEA concentration and CRC stages.

## Abbreviations

CEA: carcinoembryonic antigen
CRC: colorectal cancer
CEA-T: CEA from tumor tissues
CEA-A: CEA from tumor-adjacent normal tissues
TF-antigen: Thomsen–Friedenreich antigen
HCC: hepatocellular carcinoma
UICC: International Union Against Cancer
AJCC: American Joint Committee on Cancer
ELISA: enzyme-linked immunoadsorbent assay
CV: coefficient of variation
SEM: standard error of mean
ANOVA: one-way analysis of variance
A: tumor-adjacent normal tissue samples
T: tumor tissue samples
SD: standard deviation
M: male
F: female
C_(CEA)_: the concentration of CEA
ROC: receiver operating characteristic
AUC: area under the curve
LC: liquid chromatography
CE: capillary electrophoresis
MALDI-TOF–MS^n^: matrix-assisted laser desorption/ionization-time of flight–mass spectrometry^n^
CF: core fucosylation
Fuc: fucose
Man: mannose
Glc: glucose
GlcNAc: *N*-acetylglucosamine
GalNAc: *N*-acetylgalactosamin
Gal: galactose
NeuAc: *N*-acetylneuraminic acid
IgG: immunoglobulin G
RA: rheumatoid arthritis
