# Supplementary material for: Glycan analysis of colorectal cancer samples reveals stage-dependent changes in CEA glycosylation patterns
Source: Clin Proteomics. 2018 Mar 2;15:9. doi: 10.1186/s12014-018-9182-4 (PMC5834848; doi:10.1186/s12014-018-9182-4)
Supplement: Supplementary file 1 — Additional file 1: Table S1. Lectin used in this study. [file 12014_2018_9182_MOESM1_ESM.docx]

**Supplementary Table 1. Lectin used in this study.**

| **Abbr.** | **Ref.** | | **Specificity** | **Specificity-Cartoon** | **Source** | **Fullname/Source species** |
| --- | --- | --- | --- | --- | --- | --- |
| AAL | 1 | | Fuca1-2,3,4 | 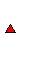 | 2 | Aurentia lectin |
| ABA | 1 | | Galb1-3GalNAc | 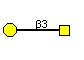 | 4 | Agaricus bisporus Lectin (Mushroom) |
| ACL ,ACA | 2 | | Galb1-3GalNAc | 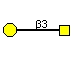 | 4 | Amaranthus caudatus Lectin (Amaranthin) |
| AMA | 4 | | Mana | 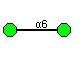 | 4 | Arum maculatum Lectin (Lords and Ladies) |
| ASA | 1 | | Mana1-3 | 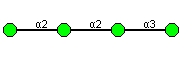 | 4 | Allium sativum Lectin (Garlic) |
| BBC | 4 | | GalNAca, GalNAcb | 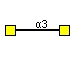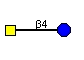 | 4 | Phaseolus vulgaris sp. Lectin (Black Bean) crude |
| BPL | 5 | | Galb1-3GalNAc, not to terminal α-GalNAc | 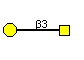 | 3 | [Bauhinia Purpurea Lectin](http://www.vectorlabs.com/products.details.asp?prodID=163) |
| CALSEPA | | 5 | Man, Glc, Glca1-4Glc | 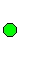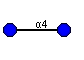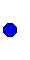 | 4 | Calystega sepiem Lectin (Hedge Bindweed Rhizomes) |
| Con A | 1 | | Man (Terminal, Branched), GlcNAc (Terminal) | 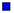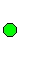 | 1 | Canavalia ensiformis, jack bean |
| CSA | 1 | | GalNAca (Terminal) | 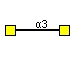 | 4 | Cytisus sessilifolius Lectin (Portugal Broom) |
| DBA | 2 | | GalNAca1-3GalNAc, GalNAca1-3Gal | 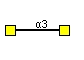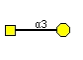 | 4 | Dolichos biflorus Lectin (Horse Gram) |
| DSL | 5 | | GlcNAc | 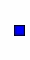 | 3 | [Datura Stramonium Lectin](http://www.vectorlabs.com/products.details.asp?prodID=181) |
| ECL | 1 | | Galb1-4GlcNAc (Terminal) | 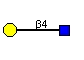 | 4 | Erythrina cristagalli Lectin (Coral Tree) |
| EEL | 1 | | GalNAcb | 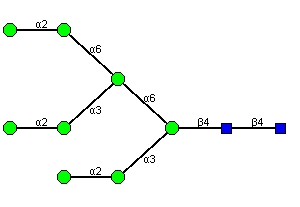 | 4 | Euonymus europaeus Lectin (Spindle Tree) |
| GHA | 1 | | Gala, GalNAca | 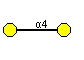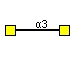 | 4 | Glechoma hederacea Lectin (ground ivy) |
| GNL | 1 | | Mana (Terminal) | 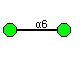 | 2 | Galanthus nivalis agglutinin (snowdrop) |
| GSL-IA4 | 5 | | GalNAca | 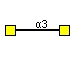 | 2 | Griffonia simplicifolia agglutinin |
| GSL-IB4,  BSLI-B4 | 2 | | Gala (Terminal) | 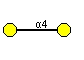 | 1 | Griffonia simplicifolia agglutinin |
| GSL-II  BSL-II | 3 | | GlcNAca, GlcNAcb | 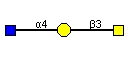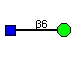 | 1 | Griffonia simplicifolia lectin |
| HHL AL | 1 | | Mana (Terminal) | 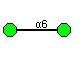 | 4 | Hippeastrum hybrid Lectin (Amaryllis bulbs) |
| HMA | 4 | | GalNAca, Fuca, Neu5Ac | 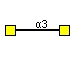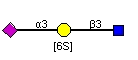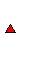 | 4 | Homarus americanus Lectin (Lobster) |
| HPA | 2 | | GalNAca (Terminal) | 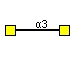 | 1 | Helix pomatia agglutinin (Roman snail, edible snail) |
| IAA | 5 | | GalNAc | 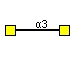 | 4 | Iberis amara Lectin |
| IRA | 4 | | GalNAca, GalNAcb | 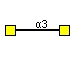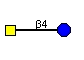 | 4 | lris hybrid Lectin (Dutch Iris) |
| Jacalin,  AIA | 1 | | Gala, Galb, GalNAca (O-linkage) | 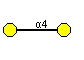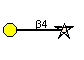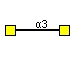 | 1 | [Jackfruit lectin (Artocarpus heterophyllus) (bread fruit tree)](http://bj.portlandpress.co.uk/bj/367/0817/bj3670817.htm) |
| LAL | 5 | | Fuc? | 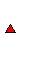 | 4 | Laburnum anagyroides Lectin (Gold Chain) |
| LCA | 5 | | Man | 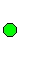 | 3 | [Lens Culinaris Agglutinin](http://www.vectorlabs.com/products.details.asp?prodID=230) |
| LEL (TL) | 5 | | GlcNAc (prefer trimer and tetramer) | 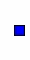 | 3 | [Lycopersicon Esculentum (Tomato) Lectin](http://www.vectorlabs.com/products.details.asp?prodID=242) |
| LTL | 1 | | Fuca1-2,3,4 | 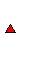 | 4 | Lotus tetragonolobus Lectin (Asparagus Pea) |
| MAA | 2 | | Neu5Aca2-3Gal | 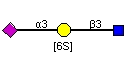 | 4 | Maackia amurensis Lectin |
| MAL-I | 5 | | Galb1-4GlcNAc | 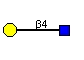 | 3 | [Maackia Amurensis Lectin I](http://www.vectorlabs.com/products.details.asp?prodID=246) |
| MAL-II | 5 | | Neu5Aca2-3 | 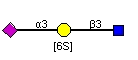 | 3 | [Maackia Amurensis Lectin II](http://www.vectorlabs.com/products.details.asp?prodID=249) |
| MNA-G | 4 | | Gala, Galb | 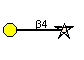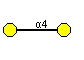 | 4 | Morniga G Lectin (black elderberry) |
| MNA-M | 4 | | Mana | 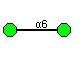 | 4 | Morniga M Lectin (black elderberry) |
| MPL | 1 | | Gala, GalNAca | 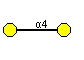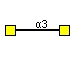 | 4 | Maclura pomifera Lectin (Osage Orange) |
| NPL NPA | 4 | | Mana? Manb? | 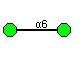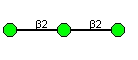 | 4 | Narcissus pseudo-narcissus Lectin (Daffodil) |
| PSA | 4 | | Mana, Glca, GlcNAca | 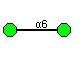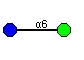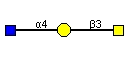 | 4 | Pisum sativum Lectin (Garden Pea) |
| PHA-E | 2 | | Galb1-4GlcNAcb1-2(Galb1-4GlcNAcb1-6)Man | 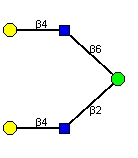 | 4 | Phaseolus vulgaris Lectin (Red Kidney Bean) |
| PHA-L | 2 | | Galb1-4GlcNAcb1-2Man | 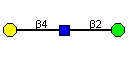 | 1 | Phaseolus vulgaris Lectin (Red Kidney Bean) |
| PNA | 1 | | Galb (Terminal) | 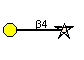 | 4 | Arachis hypogaea lectin from peanut |
| PTL I  WBA I,  Gal | 5 | | Gal | 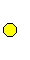 | 4 | Psophocarpus tetragonolobus Lectin (Winged Bean) |
| PTL I  WBAI ,GalNAc | 5 | | GalNAc | 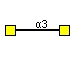 | 4 | Psophocarpus tetragonolobus Lectin (Winged Bean) |
| PWM (PWA) | 5 | | GlcNAcb1-4GlcNAc | 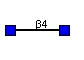 | 4 | Phytolacca americana Lectin (Pokeweed) |
| RCA-I  RCA120 | 5 | | Galb | 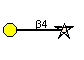 | 4 | Ricinus communis Lectin (Castor Bean) |
| SBA | 2 | | GalNAca (Terminal), Neu5Aca2-6GalNAc (Tn antigen) | 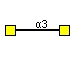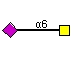 | 1 | Soybean agglutinin (*Glycine max,* soya bean) |
| SNA  EBL | 2 | | Neu5Aca2-6Gal, Neu5Aca2-6GalNAc | 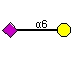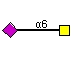 | 2 | Sambucus nigra (Elderberry Bark) |
| SNA-I | 1 | | Neu5Aca2-6Galb1-4GlcNAc, Neu5Aca2-6Galb1-4Glc | 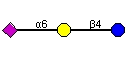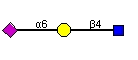 | 4 | Sambucus nigra (Elderberry Bark) |
| SSA | 5 | | GalNAc, Terminal, O-link | 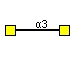 | 4 | Salvia sclarea |
| STL (PL) | 5 | | GlcNAc, Neu5Ac | 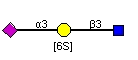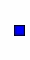 | 3 | [Solanum Tuberosum (Potato) Lectin](http://www.vectorlabs.com/products.details.asp?prodID=306) |
| TL | 4 | | α−GalNAc,β−GalNAc, GalNAc, Gal, Fucose | 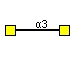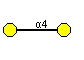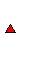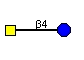 | 4 | Tulipa sp. Lectin (Tulip) |
| UDA | 1 | | GalNAcb | 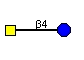 | 4 | Urtica dioica Lectin (Stinging Nettle) |
| UEA-I | 3 | | Fuca | 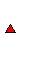 | 4 | Ulex europaeus Lectin (Gorse, Furze) |
| VFA | 1 | | Mana | 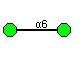 | 4 | Vicia fava Lectin (Fava Bean) |
| VVL  VVA | 4 | | GalNAca, GalNAca1-3Gal | 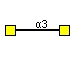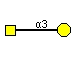 | 4 | Vicia villosa Lectin (Hairy Vetch) |
| VVA ,Man | 5 | | Man | 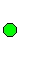 | 4 | Vicia villosa Lectin (Hairy Vetch, Mannose Specific) |
| WFA,  WFL | 4 | | GalNAca, GalNAcb | 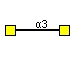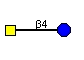 | 4 | Wisteria floribunda Lectin (Japanese Wisteria) |
| WGA | 1 | | GlcNAcb | 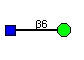 | 1 | Triticum vulgaris lectin from wheat germ |

The lectin carbohydrate specificities were summarized from 5 different references: 1, The database of CFG; 2, Hsu et. al. A lectin microarray approach for the rapid analysis of bacterial glycans. Nat Protocols. 2006;1:543–9.; 3, Wearne et. al. Use of lectins for probing differentiated human embryonic stem cells for carbohydrates. Glycobiology. 2006; 16:981–990.; 4, The lectin carbohydrate specificity chart from EY lab; 5, Manual associated with the purchased lectin from EY or Vector. The lectins used in this study were obtained from 4 different sources: 1, Molecular Probes Inc.; 2, Irwin J. Goldstein's group; 3, Vector laboratories Inc.; 4, EY Laboratories Inc.
