# Supplementary material for: Glycan analysis of colorectal cancer samples reveals stage-dependent changes in CEA glycosylation patterns
Source: Clin Proteomics. 2018 Mar 2;15:9. doi: 10.1186/s12014-018-9182-4 (PMC5834848; doi:10.1186/s12014-018-9182-4)
Supplement: Supplementary file 4 — Additional file 4: Table S2. The concentration of CEA of CRC patients; Figure S1. The relationship between CEA concentration and CRC stages. [file 12014_2018_9182_MOESM4_ESM.docx]

**Supplementary Table 2. The concentration of CEA of CRC patients:**

| **Stage** | **Code** | **CEA** (ng/ml)  (In the blood) | **CEA** (μg/ml)  (In the tumor-adjacent normal tissues) | | **CEA** (μg/ml)  (In the tumor tissues) |
| --- | --- | --- | --- | --- | --- |
| Ⅰ | 3 | 0.87 | 6.51 | 61.04 | |
|  | 4 | 4.33 | 6.70 | 40.24 | |
|  | 5 | 2.49 | 6.23 | 11.77 | |
|  | 6 | 2.50 | 3.25 | 68.91 | |
|  | 7 | 1.00 | 6.52 | 34.81 | |
|  | 8 | 4.61 | 5.45 | 34.12 | |
|  | 9 | 3.45 | 3.67 | 16.23 | |
|  | 11 | 3.49 | 3.20 | 51.35 | |
|  | 12 | 4.34 | 7.81 | 33.71 | |
|  | 13 | 2.00 | 5.86 | 14.61 | |
|  | 14 | 1.94 | 2.73 | 42.30 | |
|  | 15 | 1.67 | 7.51 | 37.50 | |
|  | 16 | 2.76 | 7.30 | 32.31 | |
|  | 17 | 0.76 | 4.83 | 26.92 | |
|  | 18 | 1.98 | 2.53 | 22.88 | |
|  | 19 | unknown | 4.25 | 16.25 | |
|  | 20 | 3.39 | 5.06 | 35.42 | |
| Ⅱ | 2 | 1.73 | 2.85 | 15.47 | |
|  | 3 | 5.86 | 9.19 | 57.93 | |
|  | 5 | 1.76 | 28.94 | 6.97 | |
|  | 6 | 7.87 | 17.30 | 31.01 | |
|  | 7 | 2.71 | 2.61 | 77.59 | |
|  | 8 | 2.60 | 3.55 | 8.89 | |
|  | 9 | 1.34 | 5.41 | 95.20 | |
|  | 11 | unknown | 5.00 | 30.08 | |
|  | 12 | 2.03 | 6.21 | 63.53 | |
|  | 13 | unknown | 7.01 | 12.19 | |
|  | 16 | 17.38 | 4.70 | 8.99 | |
|  | 17 | 5.65 | 2.77 | 117.35 | |
|  | 19 | 5.90 | 4.13 | 7.44 | |
|  | 20 | 1.66 | 2.95 | 33.41 | |
| Ⅲ | 2 | 1.86 | 5.18 | 48.68 | |
|  | 3 | unknown | 12.99 | 22.40 | |
|  | 5 | 5.38 | 4.40 | 56.42 | |
|  | 6 | 3.40 | 8.52 | 17.12 | |
|  | 8 | 9.41 | 8.28 | 55.94 | |
|  | 9 | unknown | 5.59 | 11.63 | |
|  | 14 | 2.07 | 3.74 | 10.08 | |
|  | 17 | 9.52 | 4.31 | 20.78 | |
|  | 18 | 9.12 | 4.35 | 20.12 | |
| Ⅳ | 1 | 110.20 | 4.59 | 45.40 | |
|  | 2 | 1.06 | 3.96 | 6.04 | |
|  | 3 | 29.94 | 5.32 | 123.07 | |
|  | 4 | 1.42 | 2.84 | 10.32 | |
|  | 5 | 4227 | 3.45 | 44.52 | |
|  | 6 | unknown | 3.54 | 52.89 | |
|  | 7 | 1.24 | 5.94 | 9.23 | |
|  | 8 | 16.55 | 3.69 | 18.22 | |
|  | 9 | 71.32 | 4.66 | 65.00 | |
|  | 11 | 16.49 | 6.53 | 24.25 | |
|  | 12 | 21.96 | 15.69 | 29.42 | |
|  | 13 | unknown | 3.83 | 34.02 | |
|  | 19 | 1.22 | 2.56 | 12.54 | |


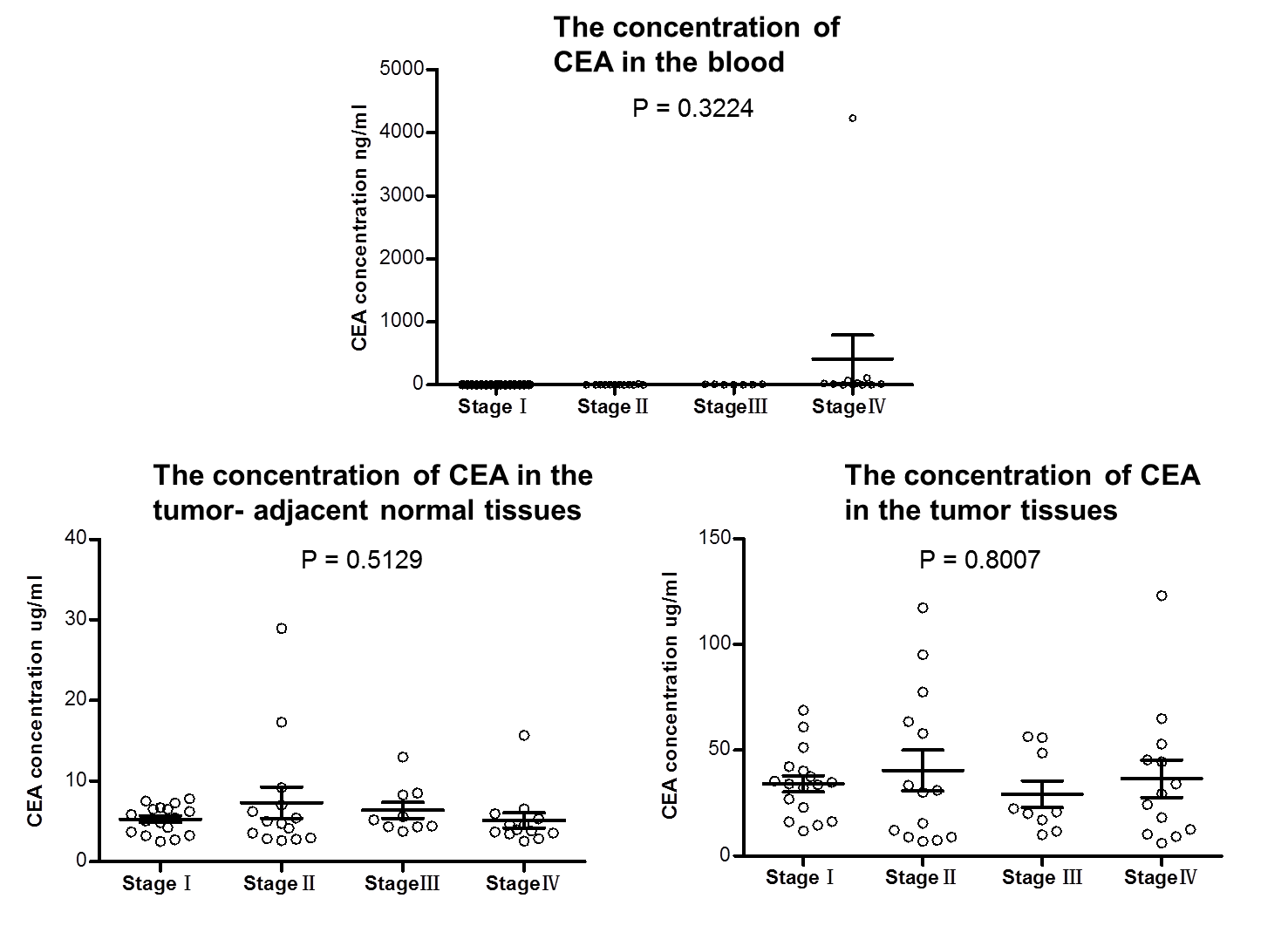


**B**

**C**

**A**

**Figure S1: The relationship between CEA concentration and CRC stages.** (A) The concentration of CEA in the blood. (B) The concentration of CEA in the tumor-adjacent normal tissues. (C) The concentration of CEA in the tumor tissues.
